# Surgical Management of Gastric Cancer: A Systematic Review

**DOI:** 10.3390/jcm10122557

**Published:** 2021-06-09

**Authors:** Lucian Mocan

**Affiliations:** 1 Department of Surgery, Iuliu Hatieganu University of Medicine and Pharmacy, RO-400012 Cluj-Napoca, Romania; lucian.mocan@umfcluj.ro or mocanlucian@yahoo.com; Tel.: +40-745-362-345; 2 Regional Institute of Gastroenterology and Hepatology, 19-21 Croitorilor Street, RO-400162 Cluj-Napoca, Romania

**Keywords:** gastric cancer, surgery, lymphadenectomy, survival

## Abstract

Gastric cancer is the fifth most common cancer worldwide, and it is responsible for 7.7% of all cancer deaths. Despite advances in the field of oncology, where radiotherapy, neo and adjuvant chemotherapy may improve the outcome, the only treatment with curative intent is represented by surgery as part of a multimodal therapy. Two concepts may be adopted in appropriate cases, neoadjuvant treatment before gastrectomy (G) or primary surgical resection followed by chemotherapy. Such an approach, combined with early detection and better screening, has led to a decrease in the overall incidence of gastric cancer. Unfortunately, malignant tumors of the stomach are often diagnosed in locally advanced or metastatic stages when the median overall survival remains poor. Surgical care in these cases must be provided by a multidisciplinary team in a high-volume center. Important surgical aspects such as optimum resection margins, surgical technique, and number of harvested lymph nodes are important factors for patient outcomes. The standardization of surgical treatment of gastric cancer in accordance with the patient’s profile is of decisive importance for a better outcome. This review aims to summarize the current standards in the surgical treatment of gastric cancer.

## 1. Introduction

Gastric cancer is the fifth most common cancer worldwide, and it is responsible for 7.7% of all cancer deaths. Although surgical treatment for gastric cancer has been considerably improved during recent decades, the mortality rate from gastric cancer is still high [1]. Statistical data show that the 5-year survival rate for patients treated with curative intent (gastric resection and lymphadenectomy) is 70% for stage I resected gastric cancer and less than 30% for stage IIB disease and beyond [2]. Most gastric tumors are adenocarcinomas [3]. Despite advances in the field of oncology, where radiotherapy, neo and adjuvant chemotherapy may improve the outcome, the only treatment with curative intent is represented by surgery as part of a multimodal therapy [4]. Two concepts may be adopted in appropriate cases, neoadjuvant treatment before gastrectomy or primary surgical resection followed by chemotherapy [5].

Genetic alterations responsible for the development and progression of gastric cancer such as cell adhesion, signal transduction, DNA methylation, and glycosylation changes may lead to early detection of gastric cancers using these biomarkers [6].

For patients with hereditary diffuse gastric cancer (HDGC), who carry a lifetime gastric cancer risk of approximately 70% in men and 56% in women, a prophylactic total gastrectomy at the age of 20 years is the procedure of choice [7]. Recently, there have been numerous sources of evidence establishing the importance of combining systemic chemotherapy with surgery in advanced gastric cancer. Given the latest results, there has been a shift in the paradigm of gastric cancer treatment with the increasing use of preoperative and postoperative chemotherapy [8].

**Figure 1:**
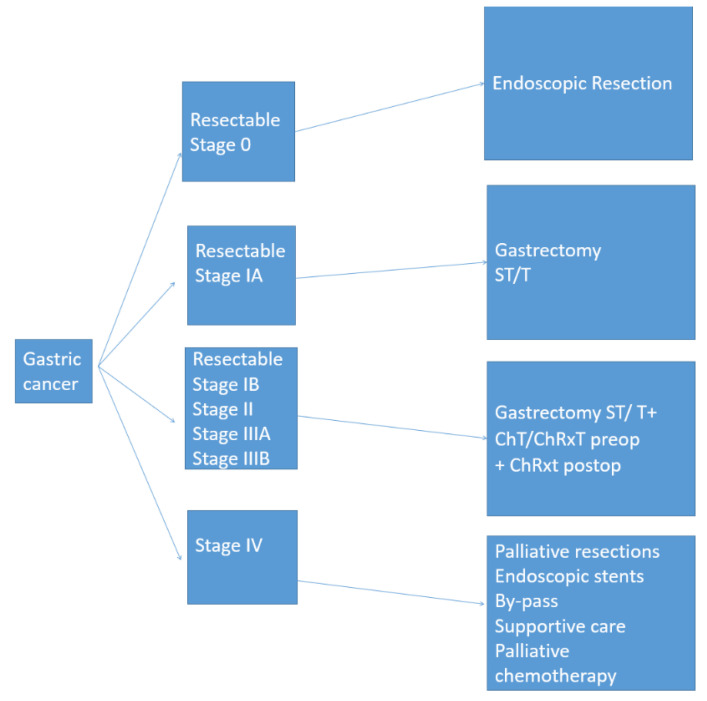
Treatment strategies of gastric cancer according to TNM stage. Stage 0: TisN0M0; stage IA: T1N0M0; stage IB: T2N0M0; stage II: T1N2M0/T2N1M0/T3N0M0; stage IIIA: T3N1M0/T4N0M0; stage IIIB: T3N2M0; stage IV: T3N1-3M0/T1-3N3M0/T1-4N0-3M1 (Tis—the mucosa; T1—submucosa;T2—muscle layer; T3—subserosa; T4—serosa/adjacent structures/N0—(0+)LN; N1—(1–2+)LN; N2—(3–6+)LN; N3—(>7+)LN/M0—no metastasis; M1—distant metastasis or carcinomatosis); LN—lymph nodes; ST—subtotal; T—total; ChT—chemotherapy; ChRxT—chemo-radiotherapy; preop—preoperative; postop—postoperative.

Unfortunately, malignant tumors of the stomach are often diagnosed in locally advanced or metastatic stages when the median overall survival remains poor [9]. Surgical care in these cases must be provided by a multidisciplinary team in a high-volume center [10]. Important surgical aspects such as optimum resection margins, surgical technique, number of harvested lymph nodes are important factors for patient outcomes. The standardization of surgical treatment of gastric cancer in accordance with the patient’s profile is of decisive importance for a better outcome (Figure 1). This review aims to summarize the current standards in the surgical treatment of gastric cancer.

## 2. Results and Discussion

### 2.1. Extent of Gastric Resection: Total Gastrectomy (TG), Subtotal Gastrectomy (SG), and Proximal Gastrectomy (PG)

The extent of surgical resection required to achieve surgical margins free of malignant cells, R0, depends on the size, location, and histological type of the tumor. The optimal length for the proximal margin is often suggested to be at least 3 to 5 cm depending on the tumor histology [11]. However, recent studies suggest that resection margins of 1 cm may be comparable in terms of survival and oncological outcome [12].

Since the standard approach for gastric cancer with any localization is total gastrectomy (TG), several studies have shown that the outcomes of patients with proximal tumors who underwent TG or proximal gastrectomy (PG) were similar in terms of the overall survival interval and disease-free interval [13]. Following these studies, it is accepted today that both procedures could be accomplished safely. Some authors suggest that distal gastrectomy can be safely performed for patients with distal lesions and TG/PG may be performed for proximal lesions [14]. The benefit of PG for the surgical treatment of proximal cancers was assessed by Harrison et al. [15], and the researchers showed that patients with proximal tumors who underwent PG resection had similar overall survival and disease-free intervals compared with patients who underwent TG resection. The only concern related to PG was represented by the increased number of patients who experienced late complications, such as esophageal reflux [16]. Several trials [17,18,19,20,21] addressed the issues of postoperative mortality, morbidity, and long-term outcome of TG versus subtotal gastrectomy (SG) for distal tumors. The wound infection, anastomotic fistula, and mortality rates were higher in the TG group compared with the SG group. However, there was no difference in the postoperative mortality rate or 5-year survival rate between the two groups (TG vs. SG). For patients who underwent radical SG, studies have shown an improved quality of life compared with the TG group [22].

The controversy continues in tumors of the cardia when radical resection requires supradiaphragmatic anastomosis [23]. There are three therapeutic options in such cases: some surgeons prefer PG, whereas others adopt either TG or esophagectomy with proximal cervical anastomosis as the ideal therapeutic option. Ito et al. [24] showed no difference between total esophagectomy, thoracic esophagogastrectomy, and abdominal esophagogastrectomy in terms of 5-year survival rates. Postoperative complications were significantly higher in patients with esophagectomy (33% vs. 11%). In these cases, for the optimal (R0) resection, >4 cm (distal) gastric margin and >6 cm esophageal margin must be considered. The actual recommendations are to avoid the transabdominal approach only for the extent of esophageal resection in tumors involving the distal esophagus and cardia. In such cases, an individualized operative approach is recommended for an optimal R0 resection [25]. For patients who have extensive linitis plastica, TG is more frequently performed [26]. Recent reports have indicated that, if R0 can be safely achieved, pylorus-preserving resection for gastric cancer should be considered because no difference in survival was reported compared with more extensive procedures [27,28].

### 2.2. Reconstruction Following Resection

The technique of anastomosis that began in 1881, when Theodor Billroth performed the first gastrectomy, has been extensively explored in numerous studies since then [29]. Although Billroth I was the method of choice for a long time, it is currently accepted that the bile reflux gastritis is best minimized by Roux-en-Y reconstruction [30]. However, until the 21st century, the general preference for TG was to use jejunum loop reconstruction; during the last two decades, Roux-en-Y reconstruction became the standard procedure worldwide [31]. The Roux-en-Y reconstruction following TG is also a preferred method of reconstruction after pancreatic and biliary resections and liver and pancreatic cysts, as well as in bariatric surgery [32].

The reconstruction using jejunal pouches has historical value, but such pouches are no longer employed because they show limited benefit. Small-bowel interposition was preferred in the past by some surgeons; today, it has less acceptance and is rarely used [33].

### 2.3. Extending Resection to Adjacent Organs

Extended resection (D2 resection with splenectomy and distal pancreatectomy) for advanced gastric cancer, initially performed by Japanese surgeons, has been a subject of debate for decades [34]. Although early reports showed improved survival [35], large prospective randomized control trials failed to report a real survival benefit. In patients with splenectomy and distal pancreatectomy, higher morbidity, higher mortality, and longer hospital stays were observed [36]. In a study published by Otsuji et al. [37], out of 128 patients who underwent TG for gastric adenocarcinoma of the middle or proximal stomach, 35.9% underwent pancreatosplenectomy (PS), 44.6% underwent splenectomy (S), and underwent 19.5% gastrectomy alone. The morbidity and mortality were higher in patients with pancreatosplenectomy, mainly due to pancreatic fistula occurrence. Five-year survival rates of 40.7% (PS), 55.9% (S), and 54.2% (G) were reported. Importantly, in multivariate analysis, PS and S alone were found to not be independent factors for survival, strongly suggesting that PS increases morbidity rates without improving survival.

A series of 353 patients who underwent extended resection of the adjacent organs (removal of transverse colon in 45%, pancreas and spleen in 42.5%, left hepatic lobe in 28.5%, and head of the pancreas in 10.5%) was published by Shchepotin et al. [38]. TG was performed in 32.9% of patients and SG in 67.1%. The lymphadenectomy was standardly performed in all patients (lymph nodes around stomach, celiac axis, hepatic artery, and proximal splenic artery). The presented data displayed a 5-year survival rate of 25% (37% for N− and 15% for N+).

Data arising from two prospective randomized control trials that do not favor gastrectomy with additional organ resection have been published [39,40]. Bonenkamp et al. [41] reported the results of extended gastrectomy on 996 patients randomized to a D1 or D2 lymph node dissection. A significant increase in postoperative complications, reoperation rates, and hospital stays was seen in patients requiring a D2 lymphadenectomy [42]. D2 with splenectomy and/or pancreatectomy was significantly responsible for this poor outcome [43]. In another paper, Kasakura et al. [44] showed that removal of an additional organ was not a factor for survival in stages II, III, and IV. We can conclude that extended resection (where R0 is feasible) of the adjacent organs can be performed by highly experienced surgeons in patients with T4 tumors.

### 2.4. Extent of Lymphadenectomy

The extent of lymphadenectomy performed along with gastrectomy has been a debated subject for decades [45,46,47,48]. The concept was first described by the Japanese Research Society for Gastric Cancer (JRSGC) in 1973 [49]. Comparisons between limited D1 (perigastric lymph nodes), extended D2 (perigastric and celiac axis lymph node stations), and D3 (perigastric, celiac axis, and para-aortic lymph node stations) lymphadenectomies have been analyzed for decades in prospective randomized trials.

Some authors suggest that the oncological benefit of extended nodal resection does not overcome the drawbacks of postoperative morbidity and mortality. Most Western surgeons consider that extended nodal dissection has no benefit for overall survival and malignant lymph nodes are prognostic indicators rather than factors of survival. Other surgeons (e.g., Japanese surgeons) think that the optimal therapy associated with better loco-regional control is radical gastrectomy with extensive lymphadenectomy [50]. These facts have also been confirmed by several experienced surgeons who performed complete D2 lymphadenectomy and showed that complications are no higher for D2 in surgeries performed by experienced surgeons; however, the 5-year survival rate because of prevention of loco-regional recurrences is significantly higher in those patients [51].

A highly cited Dutch trial was conducted by the Dutch Gastric Cancer Group from August 1989 to July 1993 [52]. The researchers randomized D1 and D2 dissection into two groups (711 patients in total). The D1 lymphadenectomy addressed perigastric lymph nodes only, and extended D2 lymphadenectomy incorporated additional clearance of celiac axis lymph nodes. Distal pancreatectomy with splenectomy was routinely performed for D2 completion. The published results of the trial showed a higher postoperative morbidity (43% vs. 4%, *p* < 0.001) and mortality (10% vs. 4%, *p* < 0.004) in the D2 lymphadenectomy group compared with the other group. Importantly, no difference in 5-year survival between the two groups (34% in D1 vs. 33% in D2) was observed. The unanimous conclusion following this trial was that routinely performed D2 lymphadenectomy in gastric cancer patients has no benefits for long-term survival.

The same Dutch Gastric Cancer Trial (DGCT) group recently reported data from a 15-year follow up after the above randomized nationwide Dutch D1/D2 trial and showed that disease-specific survival was significantly higher in patients receiving D2 versus D1 lymphadenectomy, but there was no improvement in overall survival [53].

The MRC trial, led by Alfred Cuschieri et al. [54], was a large, multicenter trial (32 surgeons) including 400 patients, who were divided into two groups. In one group, 200 patients underwent D1 dissection (lymphadenectomy within 3.0 cm of the tumor survival); in the other group, the remaining 200 patients had D2 dissection (lymphadenectomy of the omental bursa, hepatoduodenal nodes, and retroduodenal nodes for distal cancers and the splenic artery/splenic pedicle nodes for proximal cancers). In this trial, the postoperative morbidity and mortality were significantly higher in the D2 group (D2 vs. D1: 46% vs. 28%, *p* < 0.001; 13% vs. 6.5%; *p* = 0.04) The obtained results were comparable in terms of 5-year survival rates (35% for D1 resection and 33% for D2), gastric cancer-specific survival (hazard ratio (HR) = 1.05, 95% confidence interval (CI): 0.79–1.39), and recurrence-free survival (HR = 1.03, 95% CI: 0.82–1.29). Based on the findings of the trial, the authors suggested that classical Japanese D2 resection offered no survival advantage over D1 resection.

Following the criticism of the Dutch trial because of the high complication rate, an Italian phase II study [55] was proposed to clarify the importance of D2 dissection. To avoid potential bias, only surgeons with extensive experience in gastric cancer surgery were allowed to participate. In 191 patients with D2 lymphadenectomy (with spleen preservation), the authors showed almost similar morbidity rates following D1 and D2 lymph node dissection (12.0% vs. 17.9%, *p* = 0.178); there were also comparable results in terms of the 30-day postoperative mortality rates (D1 vs. D2: 3.0% vs. 2.2%, *p* = 0.72).

To assess the importance of an extended D2 (para-aortic lymph nodes) resection following gastric resection for cancer, a randomized trial was conducted by East Asia Surgical Oncology [56], in which 269 patients were divided into two groups. There were 135 patients in the D2 resection group and 134 in the D2+ para-aortic lymphadenectomy group. The authors reported comparable 5-year survival intervals between the two groups (52.6% for D2 vs. 55.0% for D2+, χ2 = 0.064; *p* = 0.80). The presented data failed to impose prophylactic para-aortic lymphadenectomy as a standard technique in gastric cancer treatment.

A significantly better disease-specific survival was observed in D2 compared with D1 lymphadenectomy in a Cochrane systematic review (Hazard Ratio 0.81, 95% CI: 0.71–0.92), although the rate of mortality was higher in the D2 group (Risk Ratio 2.02, 95% CI: 1.34–3.04). No statistically significant difference was observed in the disease-free interval between the D1 and D2 groups [57]. The Japan Clinical Oncology Group (JCOG) trial 9501 compared D2 and D3 lymphadenectomy [58]. The D3 lymphadenectomy (additional dissection of para-aortic lymph nodes) group had a higher morbidity rate, but the overall 5-year survival and local recurrence were the same between the two groups. Data analysis from the Surveillance, Epidemiology, and End-Results (SEER) database has shown that survival benefits occurred in patients with gastric resections who had >15 lymph nodes excised during gastrectomy [59]. Although D2 lymphadenectomy is not mandatory for gastric cancer treatment, it is strongly recommended. The current The National Comprehensive Cancer Network NCCN guidelines sustain that lymphadenectomy should remove at least 15 nodes to optimize oncologic outcomes in gastric cancer [60].

Anastomotic leakage, pancreatic leakage, and higher reoperation rates were associated with D2 dissection in the MRC [55] and Dutch trials [41], mainly because of inadequate surgical training in splenectomy and pancreatectomy rather than D2 itself. An Italian trial showed for the first time that D2 dissection without splenectomy and distal pancreatectomy has the same mortality and morbidity as the same surgery does for D1 dissection [61]. Routinely performed splenectomy or distal pancreatectomy may be considered efficient only when the primary tumor or metastatic lymph nodes directly invade the pancreas and spleen [62].

### 2.5. Influence of Positive Resection Margins and Re-Resection

Recent studies have shown that the incidence of positive margins following extended resection for advanced gastric cancer is around 24% [63]. This rate includes reports from pathology examinations (R1) and macroscopic validation of malignant tissue on resection margins (R2) [64].

A retrospective study was conducted by Cho et al. [65] over a 15-year period. Of the 2740 patients included, 49 (1.8%) had positive margins (29 proximal and 20 distal), and multivariate analysis identified extragastric extension and total gastrectomy as independent risk factors for positive resection margins (*p* = 0.015 and *p* = 0.014, respectively). Long-term survival was also significantly lower in patients with positive margins than in those with negative margins (*p* = 0.0028 and *p* = 0.025, respectively).

Multiple prospective randomized trials regarding the influence of the R1 margin on gastric cancer survival have been conducted in Asian and Western populations, but the results have been controversial [66,67,68]. Several authors found that positive margins are an independent risk factor for survival following gastric resection for cancer [69]. A multivariate regression analysis performed by Bickenbach [70] showed that R1 margins were associated with poor survival, but this association was only observed in patients with fewer than three positive lymph nodes or T1–2 disease. Schoenfeld et al. [68] showed that R1 margins were associated with a lower disease-free interval, but overall survival rates were comparable to those for R0. Regarding the opportunity for a re-resection following R1, Raziee et al. [69] obtained controversial results via a systematic review, but they agreed that re-resection should be performed to eliminate the R1 margin whenever feasible [71].

### 2.6. The Importance of High-Volume Centers

It has been stated in previous studies that high-volume departments are significantly associated with better survival, lower mortality and morbidity, and lower reoperation rates following resection for gastric cancer [72,73,74,75]. However, these studies have been criticized for a lack of statistical power because most have been retrospective reviews with few patient data [73,75]. Moreover, they have neglected major variables, such as recurrence rates, adjuvant therapy and long-term follow-up. An intergroup trial [76] that assessed the implementation of D2 lymphadenectomy in US hospitals showed that in 556 patients with adjuvant chemoradiotherapy and gastric resection, 54% had incomplete lymphadenectomy (less than D1), 37% had D1, and only 9% had D2. The underlying factor was the surgeon’s experience. Other studies have confirmed this point, showing that fewer than 33% of patients with curative gastrectomies had 15 or more lymph nodes removed/examined [77,78]. It has been proven using computer-based models that poor dissections of lymph nodes because of inadequate surgical techniques lead to poor survival in these patients [79,80].

An extensive meta-analysis analyzed 28 papers describing the relationship between hospital volume and surgeons’ experience; the 5-year survival showed that high-volume hospitals have fewer complications and better outcomes following gastric resection for cancer [81]. The number of procedures (gastric resection with D2) and the surgeons’ level of training and supraspecialization are key factors related to low postoperative complications, low gastrectomy-related mortality, and better five-year survival.

Studies suggest that procedure-related mortality is significantly higher in US hospitals, ranging from 5% to 13% [82]. Large statistical data examining more than 600 hospitals in the US over a 5-year period showed that the average perioperative mortality rate was 7.2%. Therefore, based on the above results and from our personal experience, we state that D2 lymphadenectomy and gastric surgery for cancer should be performed in tertiary surgical centers where surgeons are routinely performing this type of operation and have very low operative morbidity and mortality rates.

### 2.7. Laparoscopic Gastrectomy (LG)

Laparoscopic resection of gastric cancer is routinely performed worldwide [83] and has become a popular approach for treating gastric cancer in Asian countries (representing 25% of all gastric resections for cancer in Japan and South Korea) [84,85]. The surgical techniques and postoperative outcome have been well established in two prospective trials (KLASS 01 and JCOG 0703) for early gastric cancer [86,87].

A Japanese study (LOC-1) included 3630 patients with early gastric cancer treated with laparoscopic gastrectomy (LG) or open gastrectomy (OG) between 2006 and 2012. [88]. There was no significant difference in the 5-year overall survival (97.1% for LG vs. 96.3% for OG) or local recurrence rate (2.3% vs. 2.4%).

In 2006–2010, a trial developed in South Korea (KLASS-1), which involved 1400 patients with invasive distal gastric cancer limited to the submucosa, analyzed the feasibility of laparoscopic distal gastrectomy [88]. The LG group had a lower morbidity rate (13% vs. 20%) and lower wound infections (3.6% vs. 7%) compared with the OG group. Major intra-abdominal complications and perioperative mortality rates were similar between the two groups. The overall 5-year survival rate was comparable between the two groups (LG: 94.2%; OG: 93.3%), as were the cancer-specific survival rates (LG: 97.1%; OG: 97.2 percent) [89].

LG is a complicated procedure that requires training and experience [90,91]; it also necessitates support from the staff and hospital. The technical pitfalls make LG controversial for the resection of locally advanced tumors, mainly because of concerns regarding the R0 acquisition and adequate D2 dissection [92]. However, patients with LGs report a better quality of life in the early postoperative period. Ultimately, patients with invasive gastric cancer that invades no more deeply than the submucosa, regardless of lymph node metastasis (T1, any N, M0), and who are free of significant cardiopulmonary diseases, obesity, and previous upper abdominal surgery, are most suitable for LG.

## 3. Conclusions

The standardization of surgical resection in accordance with tumor stage is of decisive importance for a better outcome. As gastrectomy and adequate lymph node resection may be challenging, the treatment must be provided by a multidisciplinary team in a high-volume center. Since neo and adjuvant chemotherapy improve the outcome, multimodal therapy is the treatment of choice in stage IB and above. If R0 is technically feasible, distal gastrectomy can be safely performed for patients with distal lesions while TG/PG may be performed for proximal lesions. Splenectomy or distal pancreatectomy should not be performed as part of D2 lymphadenectomy and may be considered only when the primary tumor or metastatic lymph nodes directly invade the pancreas and spleen. As surgical centers with higher volume have very low operative morbidity and mortality rates, patients proposed for D2 lymphadenectomy and gastric resection for cancer should be referred to these hospitals. Surgeons should perform re-resection to eliminate the R1 margin whenever this is feasible. Laparoscopic gastrectomy may be performed by experienced surgeons with no compromise in surgical principles.

## Data Availability

Not applicable.

## References

[1] Suh Y., Lee J., Woo H., Shin D., Kong S., Lee H., Shin A., Yang H. (2020). National Cancer Screening Program for Gastric Cancer in Korea: Nationwide Treatment Benefit and Cost. Cancer.

[2] Petrillo A., Smyth E.C. (2020). Multimodality Treatment for Localized Gastric Cancer: State of the Art and New Insights. Curr. Opin. Oncol..

[3] Japanese Gastric Cancer Association (2021). Japanese Gastric Cancer Treatment Guidelines 2018. Gastric Cancer.

[4] Song Z., Wu Y., Yang J., Yang D., Fang X. (2017). Progress in the Treatment of Advanced Gastric Cancer. Tumor Biol..

[5] Tokunaga M., Sato Y., Nakagawa M., Aburatani T., Matsuyama T., Nakajima Y., Kinugasa Y. (2020). Perioperative Chemotherapy for Locally Advanced Gastric Cancer in Japan: Current and Future Perspectives. Surg. Today.

[6] Zheng L., Wang L., Ajani J., Xie K. (2004). Molecular Basis of Gastric Cancer Development and Progression. Gastric Cancer.

[7] Muir J., Aronson M., Esplen M., Pollett A., Swallow C.J. (2016). Prophylactic Total Gastrectomy: A Prospective Cohort Study of Long-Term Impact on Quality of Life. J. Gastrointest. Surg..

[8] Ilson D.H. (2018). Advances in the Treatment of Gastric Cancer. Curr. Opin. Gastroenterol..

[9] Bernards N., Creemers G., Nieuwenhuijzen G., Bosscha K., Pruijt J., Lemmens V. (2013). No Improvement in Median Survival for Patients with Metastatic Gastric Cancer Despite Increased use of Chemotherapy. Ann. Oncol..

[10] Nelen S., Heuthorst L., Verhoeven R., Polat F., Kruyt P.M., Reijnders K., Ferenschild F., Bonenkamp J., Rutter J., de Wilt J. (2017). Impact of Centralizing Gastric Cancer Surgery on Treatment, Morbidity, and Mortality. J. Gastrointest. Surg..

[11] Kumazu Y., Hayashi T., Yoshikawa T., Yamada T., Hara K., Shimoda Y., Nakazono M., Nagasawa S., Shiozawa M., Morinaga S. (2020). Risk Factors Analysis and Stratification for Microscopically Positive Resection Margin in Gastric Cancer Patients. BMC Surg..

[12] Kim A., Kim B.S., Yook J.H., Kim B.S. (2020). Optimal Proximal Resection Margin Distance for Gastrectomy in Advanced Gastric Cancer. World J. Gastroenterol..

[13] Qi J., Zhang P., Wang Y., Chen H., Li Y. (2016). Does Total Gastrectomy Provide Better Outcomes than Distal Subtotal Gastrectomy for Distal Gastric Cancer? A Systematic Review and Meta-Analysis. PLoS ONE.

[14] Sugoor P., Shah S., Dusane R., Desouza A., Goel M., Shrikhande S.V. (2016). Proximal Gastrectomy Versus Total Gastrectomy for Proximal Third Gastric Cancer: Total Gastrectomy is Not always Necessary. Langenbeck’s Arch. Surg..

[15] Harrison L.E., Karpeh M.S., Brennan M.F. (1998). Total Gastrectomy is Not Necessary for Proximal Gastric Cancer. Surgery.

[16] Hsu C., Chen C., Hsieh Y., Hsia J., Shai S., Kao C. (1997). Esophageal Reflux After Total Or Proximal Gastrectomy in Patients with Adenocarcinoma of the Gastric Cardia. Am. J. Gastroenterol..

[17] Park S., Chung H.Y., Lee S.S., Kwon O., Yu W. (2014). Serial Comparisons of Quality of Life After Distal Subtotal Or Total Gastrectomy: What are the Rational Approaches for Quality of Life Management?. J. Gastric Cancer.

[18] Gouzi J.L., Huguier M., Fagniez P.L., Launois B., Flamant Y., Lacaine F., Paquet J.C., Hay J.M. (1989). Total Versus Subtotal Gastrectomy for Adenocarcinoma of the Gastric Antrum. A French Prospective Controlled Study. Ann. Surg..

[19] Robertson C.S., Chung S.C., Woods S.D., Griffin S.M., Raimes S.A., Lau J.T., Li A.K. (1994). A Prospective Randomized Trial Comparing R1 Subtotal Gastrectomy with R3 Total Gastrectomy for Antral Cancer. Ann. Surg..

[20] Bozzetti F., Marubini E., Bonfanti G., Miceli R., Piano C., Crose N., Gennari L. (1997). Total Versus Subtotal Gastrectomy: Surgical Morbidity and Mortality Rates in a Multicenter Italian Randomized Trial. the Italian Gastrointestinal Tumor Study Group. Ann. Surg..

[21] De Manzoni G., Verlato G., Roviello F., Di Leo A., Marrelli D., Morgagni P., Pasini F., Saragoni L., Tomezzoli A. (2003). Subtotal Versus Total Gastrectomy for T3 Adenocarcinoma of the Antrum. Gastric Cancer.

[22] Diaz De Liano A., Oteiza Martinez F., Ciga M., Aizcorbe M., Cobo F., Trujillo R. (2003). Impact of Surgical Procedure for Gastric Cancer on Quality of Life. Br. J. Surg..

[23] Brennan M.F. (2005). Current Status of Surgery for Gastric Cancer: A Review. Gastric Cancer.

[24] Ito H., Clancy T.E., Osteen R.T., Swanson R.S., Bueno R., Sugarbaker D.J., Ashley S.W., Zinner M.J., Whang E.E. (2004). Adenocarcinoma of the Gastric Cardia: What is the Optimal Surgical Approach?. J. Am. Coll. Surg..

[25] Barbour A.P., Rizk N.P., Gonen M., Tang L., Bains M.S., Rusch V.W., Coit D.G., Brennan M.F. (2007). Adenocarcinoma of the Gastroesophageal Junction: Influence of Esophageal Resection Margin and Operative Approach on Outcome. Ann. Surg..

[26] Xiao H., Ma M., Xiao Y., Ouyang Y., Tang M., Zhou K., Hong Y., Tang B., Zuo C. (2017). Incomplete Resection and Linitis Plastica are Factors for Poor Survival After Extended Multiorgan Resection in Gastric Cancer Patients. Sci. Rep..

[27] Mao X., Xu X., Zhu H., Ji C., Lu X., Wang B. (2020). A Comparison between Pylorus-Preserving and Distal Gastrectomy in Surgical Safety and Functional Benefit with Gastric Cancer: A Systematic Review and Meta-Analysis. World J. Surg. Oncol..

[28] Bueno J.A.D., Park Y., Ahn S., Park D.J., Kim H. (2018). Function-Preserving Surgery in Gastric Cancer. J. Minim. Invasive Surg..

[29] Hu Y., Zaydfudim V.M. (2020). Quality of Life After Curative Resection for Gastric Cancer: Survey Metrics and Implications of Surgical Technique. J. Surg. Res..

[30] Hirao M., Takiguchi S., Imamura H., Yamamoto K., Kurokawa Y., Fujita J., Kobayashi K., Kimura Y., Mori M., Doki Y. (2013). Comparison of Billroth I and Roux-En-Y Reconstruction After Distal Gastrectomy for Gastric Cancer: One-Year Postoperative Effects Assessed by a Multi-Institutional RCT. Ann. Surg. Oncol..

[31] Kimura Y., Mikami J., Yamasaki M., Hirao M., Imamura H., Fujita J., Takeno A., Matsuyama J., Kishi K., Hirao T. (2021). Comparison of 5-year Postoperative Outcomes After Billroth I and Roux-en-Y Reconstruction Following Distal Gastrectomy for Gastric Cancer: Results from a multi-institutional Randomized Controlled Trial. Ann. Gastroenterol. Surg..

[32] Angrisani L., Santonicola A., Iovino P., Formisano G., Buchwald H., Scopinaro N. (2015). Bariatric Surgery Worldwide 2013. Obes. Surg..

[33] Shen J., Ma X., Yang J., Zhang J. (2020). Digestive Tract Reconstruction Options After Laparoscopic Gastrectomy for Gastric Cancer. World J. Gastrointest. Oncol..

[34] Kano Y., Ohashi M., Ida S., Kumagai K., Makuuchi R., Sano T., Hiki N., Nunobe S. (2020). Therapeutic Value of Splenectomy to Dissect Splenic Hilar Lymph Nodes for Type 4 Gastric Cancer Involving the Greater Curvature, Compared with Other Types. Gastric Cancer.

[35] Maruyama K., Okabayashi K., Kinoshita T. (1987). Progress in Gastric Cancer Surgery in Japan and its Limits of Radicality. World J. Surg..

[36] Lo S., Wu C., Shen K., Hsieh M., Lui W. (2002). Higher Morbidity and Mortality After Combined Total Gastrectomy and Pancreaticosplenectomy for Gastric Cancer. World J. Surg..

[37] Otsuji E., Yamaguchi T., Sawai K., Okamoto K., Takahashi T. (1999). Total Gastrectomy with Simultaneous Pancreaticosplenectomy Or Splenectomy in Patients with Advanced Gastric Carcinoma. Br. J. Cancer.

[38] Shchepotin I.B., Chorny V.A., Nauta R.J., Shabahang M., Buras R.R., Evans S.R. (1998). Extended Surgical Resection in T4 Gastric Cancer. Am. J. Surg..

[39] Bonenkamp J., Hermans J., Sasako M., Welvaart K., Songun I., Meyer S., Plukker J., Van Elk P., Obertop H., Gouma D. (1999). Extended Lymph-Node Dissection for Gastric Cancer. N. Engl. J. Med..

[40] Weeden S., Cuschieri A., Fielding J., Bancewicz J., Craven J., Joypaul V., Sydes M., Fayers P. (1999). Patient Survival After D1 and D2 Resections for Gastric Cancer: Long-Term Results of the UK Medical Research Council (MRC) Randomised Surgical Trial. Br. J. Cancer..

[41] Bonenkamp J., Songun I., Welvaart K., van de Velde C., Hermans J., Sasako M., Plukker J., van Elk P., Obertop H., Gouma D. (1995). Randomised Comparison of Morbidity After D1 and D2 Dissection for Gastric Cancer in 996 Dutch Patients. Lancet.

[42] Seevaratnam R., Bocicariu A., Cardoso R., Mahar A., Kiss A., Helyer L., Law C., Coburn N. (2012). A Meta-Analysis of D1 Versus D2 Lymph Node Dissection. Gastric Cancer.

[43] Yamamoto M., Baba H., Kakeji Y., Endo K., Ikeda Y., Toh Y., Kohnoe S., Okamura T., Maehara Y. (2004). Postoperative morbidity/mortality and Survival Rates After Total Gastrectomy, with splenectomy/pancreaticosplenectomy for Patients with Advanced Gastric Cancer. Hepatogastroenterology.

[44] Kasakura Y., Fujii M., Mochizuki F., Kochi M., Kaiga T. (2000). Is there a Benefit of Pancreaticosplenectomy with Gastrectomy for Advanced Gastric Cancer?. Am. J. Surg..

[45] Elmessiry M.M., El-Fayoumi T.A., Fayed H.M., Gebaly A.A., Mohamed E.A. (2020). Operative and Oncological Outcomes After D2 Versus D1 Gastrectomy of Operable Gastric Cancer: An Observational Study. J. Gastrointest. Cancer.

[46] Degiuli M., Reddavid R., Tomatis M., Ponti A., Morino M., Sasako M., Rebecchi F., Garino M., Vigano L., Scaglione D. (2021). D2 Dissection Improves Disease-Specific Survival in Advanced Gastric Cancer Patients: 15-Year Follow-Up Results of the Italian Gastric Cancer Study Group D1 Versus D2 Randomised Controlled Trial. Eur. J. Cancer.

[47] Kung C., Tsai J., Lundell L., Johansson J., Nilsson M., Lindblad M. (2020). Nationwide Study of the Impact of D2 Lymphadenectomy on Survival After Gastric Cancer Surgery. BJS Open.

[48] Oh S.E., Seo J.E., An J.Y., Choi M., Sohn T.S., Bae J.M., Kim S., Lee J.H. (2021). Compliance with D2 Lymph Node Dissection in Reduced-Port Totally Laparoscopic Distal Gastrectomy in Patients with Gastric Cancer. Sci. Rep..

[49] Murakami T. (1973). The General Rules for the Gastric Cancer Study in Surgery. Jpn. J. Surg..

[50] Faiz Z., Hayashi T., Yoshikawa T. (2021). Lymph Node Dissection for Gastric Cancer: Establishment of D2 and the Current Position of Splenectomy in Europe and Japan. Eur. J. Surg. Oncol..

[51] Sasako M. (2003). Principles of Surgical Treatment for Curable Gastric Cancer. J. Clin. Oncol..

[52] Hartgrink H., Van de Velde C., Putter H., Bonenkamp J., Meershoek-Klein Kranenbarg E., Songun I., Welvaart K., Van Krieken J., Meijer S., Plukker J. (2004). Extended Lymph Node Dissection for Gastric Cancer: Who may Benefit? Final Results of the Randomized Dutch Gastric Cancer Group Trial. J. Clin. Oncol..

[53] Songun I., Putter H., Kranenbarg E.M., Sasako M., van de Velde C.J.H. (2010). Surgical Treatment of Gastric Cancer: 15-Year Follow-Up Results of the Randomised Nationwide Dutch D1D2 Trial. Lancet Oncol..

[54] Cuschieri A., Weeden S., Fielding J., Bancewicz J., Craven J., Joypaul V., Sydes M. (1999). Patient Survival After D 1 and D 2 Resections for Gastric Cancer: Long-Term Results of the MRC Randomized Surgical Trial. Br. J. Cancer.

[55] Degiuli M., Sasako M., Calgaro M., Garino M., Rebecchi F., Mineccia M., Scaglione D., Andreone D., Ponti A., Calvo F. (2004). Morbidity and Mortality After D1 and D2 Gastrectomy for Cancer: Interim Analysis of the Italian Gastric Cancer Study Group (IGCSG) Randomised Surgical Trial. Eur. J. Surg. Oncol. (EJSO).

[56] Yonemura Y., Wu C., Fukushima N., Honda I., Bandou E., Kawamura T., Kamata T., Kim B., Matsuki N., Sawa T. (2008). Randomized Clinical Trial of D2 and Extended Paraaortic Lymphadenectomy in Patients with Gastric Cancer. Int. J. Clin. Oncol..

[57] Mocellin S., McCulloch P., Kazi H., Gama-Rodrigues J.J., Yuan Y., Nitti D. (2015). Extent of Lymph Node Dissection for Adenocarcinoma of the Stomach. Cochrane Database Syst. Rev..

[58] Fujimura T., Nakamura K., Oyama K., Funaki H., Fujita H., Kinami S., Ninomiya I., Fushida S., Nishimura G., Kayahara M. (2009). Selective Lymphadenectomy of Para-Aortic Lymph Nodes for Advanced Gastric Cancer. Oncol. Rep..

[59] Schwarz R.E., Smith D.D. (2007). Clinical Impact of Lymphadenectomy Extent in Resectable Gastric Cancer of Advanced Stage. Ann. Surg. Oncol..

[60] Ye J., Ren Y., Dai W., Chen J., Cai S., Tan M., He Y., Yuan Y. (2019). Does Lymphadenectomy with at Least 15 Perigastric Lymph Nodes Retrieval Promise an Improved Survival for Gastric Cancer: A Retrospective Cohort Study in Southern China. J. Cancer.

[61] Degiuli M., Sasako M., Ponti A. (2010). Morbidity and Mortality in the Italian Gastric Cancer Study Group Randomized Clinical Trial of D1 Versus D2 Resection for Gastric Cancer. Br. J. Surg..

[62] Saka M., Morita S., Fukagawa T., Katai H. (2011). Present and Future Status of Gastric Cancer Surgery. Jpn. J. Clin. Oncol..

[63] Jiang Z., Cai Z., Yin Y., Shen C., Huang J., Yin Y., Zhang B. (2020). Impact of Surgical Margin Status on the Survival Outcome After Surgical Resection of Gastric Cancer: A Protocol for Systematic Review and Meta-Analysis. BMJ Open.

[64] Sun Z., Li D., Wang Z., Huang B., Xu Y., Li K., Xu H. (2009). Prognostic Significance of Microscopic Positive Margins for Gastric Cancer Patients with Potentially Curative Resection. Ann. Surg. Oncol..

[65] Cho B.C., Jeung H.C., Choi H.J., Rha S.Y., Hyung W.J., Cheong J.H., Noh S.H., Chung H.C. (2007). Prognostic Impact of Resection Margin Involvement After Extended (D2/D3) Gastrectomy for Advanced Gastric Cancer: A 15-year Experience at a Single Institute. J. Surg. Oncol..

[66] Bissolati M., Desio M., Rosa F., Rausei S., Marrelli D., Baiocchi G.L., De Manzoni G., Chiari D., Guarneri G., Pacelli F. (2017). Risk Factor Analysis for Involvement of Resection Margins in Gastric and Esophagogastric Junction Cancer: An Italian Multicenter Study. Gastric Cancer.

[67] Kim Y., Squires M.H., Poultsides G.A., Fields R.C., Weber S.M., Votanopoulos K.I., Kooby D.A., Worhunsky D.J., Jin L.X., Hawkins W.G. (2017). Impact of Lymph Node Ratio in Selecting Patients with Resected Gastric Cancer for Adjuvant Therapy. Surgery.

[68] Schoenfeld J.D., Wo J.Y., Mamon H.J., Kwak E.L., Mullen J.T., Enzinger P.Z., Blaskowsky L.S., Ryan D.P., Hong T.S. (2016). The Impact of Positive Margins on Outcome among Patients with Gastric Cancer Treated with Radiation. Am. J. Clin. Oncol..

[69] Raziee H.R., Cardoso R., Seevaratnam R., Mahar A., Helyer L., Law C., Coburn N. (2012). Systematic Review of the Predictors of Positive Margins in Gastric Cancer Surgery and the Effect on Survival. Gastric Cancer.

[70] Bickenbach K., Gonen M., Strong V., Brennan M., Coit D. (2013). Association of Positive Transection Margins with Gastric Cancer Survival and Local Recurrence. Ann. Surg. Oncol..

[71] Hasbahceci M. (2020). Comment on “Dealing with the Gray Zones in the Management of Gastric Cancer: The Consensus Statement of the Istanbul Group”. Turk. J. Gastroenterol..

[72] Ramos M.F.K.P., Pereira M.A., Yagi O.K., Dias A.R., Charruf A.Z., Oliveira R.J.d., Zaidan E.P., Zilberstein B., Ribeiro-Júnior U., Cecconello I. (2018). Surgical Treatment of Gastric Cancer: A 10-Year Experience in a High-Volume University Hospital. Clinics.

[73] Hansson L.E., Sparen P., Nyren O. (1999). Survival in Stomach Cancer is Improving: Results of a Nationwide Population-Based Swedish Study. Ann. Surg..

[74] Finlayson E.V., Goodney P.P., Birkmeyer J.D. (2003). Hospital Volume and Operative Mortality in Cancer Surgery: A National Study. Arch. Surg..

[75] Wainess R.M., Dimick J.B., Upchurch G.R., Cowan J.A., Mulholland M.W. (2003). Epidemiology of Surgically Treated Gastric Cancer in the United States, 1988–2000. J. Gastrointest. Surg..

[76] Hundahl S.A., Macdonald J.S., Benedetti J., Fitzsimmons T. (2002). Surgical Treatment Variation in a Prospective, Randomized Trial of Chemoradiotherapy in Gastric Cancer: The Effect of Undertreatment. Ann. Surg. Oncol..

[77] Baxter N.N., Tuttle T.M. (2005). Inadequacy of Lymph Node Staging in Gastric Cancer Patients: A Population-Based Study. Ann. Surg. Oncol..

[78] Bilimoria K.Y., Talamonti M.S., Wayne J.D., Tomlinson J.S., Stewart A.K., Winchester D.P., Ko C.Y., Bentrem D.J. (2008). Effect of Hospital Type and Volume on Lymph Node Evaluation for Gastric and Pancreatic Cancer. Arch. Surg..

[79] Kampschöer G., Maruyama K., Van de Velde C., Sasako M., Kinoshita T., Okabayashi K. (1989). Computer Analysis in Making Preoperative Decisions: A Rational Approach to Lymph Node Dissection in Gastric Cancer Patients. J. Br. Surg..

[80] Bollschweiler E., Boettcher K., Hoelscher A., Sasako M., Kinoshita T., Maruyama K., Siewert J. (1992). Preoperative Assessment of Lymph Node Metastases in Patients with Gastric Cancer: Evaluation of the Maruyama Computer Program. J. Br. Surg..

[81] Mahar A.L., McLeod R.S., Kiss A., Paszat L., Coburn N.G. (2012). A Systematic Review of the Effect of Institution and Surgeon Factors on Surgical Outcomes for Gastric Cancer. J. Am. Coll. Surg..

[82] Wanebo H.J., Kennedy B.J., Chmiel J., Steele G., Winchester D., Osteen R. (1993). Cancer of the Stomach. A Patient Care Study by the American College of Surgeons. Ann. Surg..

[83] Hoshino N., Murakami K., Hida K., Hisamori S., Tsunoda S., Obama K., Sakai Y. (2020). Robotic Versus Laparoscopic Surgery for Gastric Cancer: An Overview of Systematic Reviews with Quality Assessment of Current Evidence. Updates Surg..

[84] Son S.Y., Kim H.H. (2014). Minimally Invasive Surgery in Gastric Cancer. World J. Gastroenterol..

[85] Hyung W.J., Yang H., Park Y., Lee H., An J.Y., Kim W., Kim H., Kim H., Ryu S.W., Hur H. (2020). Long-Term Outcomes of Laparoscopic Distal Gastrectomy for Locally Advanced Gastric Cancer: The KLASS-02-RCT Randomized Clinical Trial. J. Clin. Oncol..

[86] Kim H., Hyung W.J., Cho G.S., Kim M.C., Han S., Kim W., Ryu S., Lee H., Song K.Y. (2010). Morbidity and Mortality of Laparoscopic Gastrectomy Versus Open Gastrectomy for Gastric Cancer: An Interim report—A Phase III Multicenter, Prospective, Randomized Trial (KLASS Trial). Ann. Surg..

[87] Katai H., Sasako M., Fukuda H., Nakamura K., Hiki N., Saka M., Yamaue H., Yoshikawa T., Kojima K. (2010). Safety and Feasibility of Laparoscopy-Assisted Distal Gastrectomy with Suprapancreatic Nodal Dissection for Clinical Stage I Gastric Cancer: A Multicenter Phase II Trial (JCOG 0703). Gastric Cancer.

[88] Honda M., Hiki N., Kinoshita T., Yabusaki H., Abe T., Nunobe S., Terada M., Matsuki A., Sunagawa H., Aizawa M. (2016). Long-Term Outcomes of Laparoscopic Versus Open Surgery for Clinical Stage I Gastric Cancer: The LOC-1 Study. Ann. Surg..

[89] Kim H., Han S., Kim M., Kim W., Lee H., Ryu S.W., Cho G.S., Kim C.Y., Yang H., Park D.J. (2019). Effect of Laparoscopic Distal Gastrectomy Vs Open Distal Gastrectomy on Long-Term Survival among Patients with Stage I Gastric Cancer: The KLASS-01 Randomized Clinical Trial. JAMA Oncol..

[90] Uyama I., Suda K., Satoh S. (2013). Laparoscopic Surgery for Advanced Gastric Cancer: Current Status and Future Perspectives. J. Gastric Cancer.

[91] Yu J., Hu Y., Chen T., Mou T., Cheng X., Li G. (2013). Laparoscopic Distal Gastrectomy with D2 Dissection for Advanced Gastric Cancer. Chin. J. Cancer Res..

[92] Caruso S., Patriti A., Roviello F., De Franco L., Franceschini F., Coratti A., Ceccarelli G. (2016). Laparoscopic and Robot-Assisted Gastrectomy for Gastric Cancer: Current Considerations. World J. Gastroenterol..

